# Poorer patient-reported knee function and quality of life, but not activity level, after revision ACL reconstruction compared with primary ACL reconstruction: a matched-pair analysis with a minimum 5-year follow-up

**DOI:** 10.1186/s12891-023-06954-1

**Published:** 2023-10-23

**Authors:** Firathan Koca, Anders Stålman, Cornelia Vestberg, Riccardo Cristiani, Anne Fältström

**Affiliations:** 1 Capio Artro Clinic, FIFA Medical Center of Excellence, Department of Molecular Medicine and Surgery, Stockholm Sports Trauma Research Center, Karolinska Institutet, Stockholm, Sweden; 2 Unit of Physiotherapy, Department of Health, Medicine and Caring Sciences, Linköping University, Linköping, Sweden; 3 Region Jönköping County, Rehabilitation Centre, Ryhov County Hospital, Jönköping, SE-551 85 Sweden

**Keywords:** Anterior cruciate ligament, Reconstruction, Revision, Re-injury, Return to sport, Second knee injury, Re-rupture, Quality of life

## Abstract

**Background:**

An appropriate method for comparing knee function and activity level between patients with primary and revision anterior cruciate ligament reconstruction (ACLR) is to perform a matched-group analysis. The aim was to assess and compare knee function, knee-related quality of life and activity level between patients with revision ACLR and primary ACLR at a minimum of 5 years of follow-up.

**Methods:**

Patients aged ≤ 40 years old who underwent revision ACLR between 2010 and 2015 and a matched control group (primary ACLR) (1:1) with age ± 2 years, year of ACLR, sex, and pre-injury sport and Tegner Activity Scale (TAS) were retrospectively identified in our clinic database. The preoperative Knee injury and Osteoarthritis Outcome Score (KOOS) and surgical data were extracted and analyzed. Patients were mailed KOOS and EQ-5D questionnaires at a minimum of 5-years after revision ACLR. Study-specific questions about knee function, limitation in sport, satisfaction, and activity level according to the TAS (all scales of 1–10, 10 best) were also asked by telephone.

**Results:**

Seventy-eight patients with a revision ACLR (mean age ± SD, 29.9 ± 6.0 years) matched with seventy-eight patients with a primary ACLR (30.2 ± 5.8 years) were included. The follow-up for the revision ACLR group was 7.0 ± 1.5 years and for the primary ACLR group 7.7 ± 1.6 years. The revision ACLR group reported poorer KOOS scores in all subscales (*p* < 0.05) except the Symptoms subscale, poorer EQ-5D VAS (mean 79.2 ± 20.1 vs 86.0 ± 20.1, *p* = 0.012), and less satisfaction with current knee function (median 7 (6–8) vs 8 (7–9), *p* < 0.001). Patients with revision ACLR also experienced greater limitation in sports (median 7 (4–8) vs 8 (6–9), *p* < 0.001). There were no significant differences in the EQ-5D (mean 0.86 ± 0.17 vs 0.89 ± 0.11, *p* = 0.427), activity level (median 2 (2–5) vs 4 (2–7)*, p* = 0.229), or satisfaction with activity level (median 8 (5–9) vs 8 (6–10), *p* = 0.281) between the groups.

**Conclusions:**

At a minimum 5-year follow-up, the revision ACLR group reported poorer knee function and quality of life, less satisfaction with knee function and a greater limitation in sports but no differences in activity level and satisfaction with activity level compared with the primary ACLR group.

## Introduction

Anterior cruciate ligament (ACL) reconstruction (ACLR) is often recommended after an ACL injury in active and young patients who wish to return to pivoting sports [1]. However, a serious complication after ACLR is graft rupture, where the risk is high, especially after returning to high knee-demanding sports like football [2, 3]. Patients expect to a high degree to return to sport (RTS) at a similar level as before their injury, after both primary and revision ACLR (88% and 63% respectively) [4]. However, on average, after a primary ACLR, 65% return to their preinjury activity level [5], while the corresponding figure after a revision ACLR is reported to be 46%-62% [6, 7]. When analysing the same patients after primary ACLR and after revision ACLR, the RTS rate was 4–15% lower after revision ACLR [7, 8].

Knee function and knee-related quality of life could be negatively affected after primary and revision ACLR [8–12]. Ideal methods for comparing the results of primary and revision ACLR are either to compare the same patients after primary ACLR and then after revision ACLR or to perform a matched-group analysis. A recent study [7] showed that the same patients report inferior postoperative Knee injury and Osteoarthritis Outcome Score (KOOS) subscale scores after revision ACLR compared with primary ACLR at one year follow-up. Another recent matched-group control study (matching variables: age, sex, body mass index, and generalised hypermobility) found that patients with revision ACLR and primary ACLR reported similar scores on the Lysholm score and Tegner Activity Scale (TAS) at a 3–5-year follow-up but a significantly lower RTS rate after revision ACLR [13]. However, long-term follow-up (> 5 years) studies comparing differences in knee function, activity level and satisfaction with activity level within and between matched patients with primary and revision ACLR are lacking. The purpose of this study was to assess and compare patient-reported knee-related quality of life, activity level and satisfaction with knee function and activity level among patients who underwent revision ACLR and a control group consisting of matched patients who underwent primary ACLR at a minimum 5-year follow-up period. A second aim was to compare preoperative and postoperative KOOS subscale scores within patients with revision ACLR and the matched controls. The hypothesis was that patients who underwent revision ACLR would have inferior patient-reported knee function, activity level, and satisfaction compared with those of patients with primary ACLR.

## Methods

### Study design

The study has an observational cross-sectional design. Ethical approval was granted by the Swedish Ethical Review Authority (Dnr 2016/1613–31 and 2020–01451) and followed the principles of the Declaration of Helsinki. Participants received oral and written information about the study.

### Participants

Patients were retrospectively identified in our local database at Capio Artro Clinic, Stockholm, Sweden. The database encompasses comprehensive data regarding patient demographics, surgical details, and patient-reported outcome measurements as the preoperative KOOS [14] and the TAS [15]. Patients who were 40 years of age or younger at the time of their second ACL injury and underwent revision ACLR between 2010 and 2015 were identified as the revision ACLR group. These patients were then contacted for follow-up between June 2020 and May 2022, ensuring a minimum follow-up period of 5 years.

Patients who were not active in any sport at any level at the time of their ACL injury, had a bilateral ACLR or underwent simultaneous reconstruction of other ligaments (such as collateral ligaments or posterior cruciate ligament) during either primary or revision ACLR were excluded from the study. Concomitant injuries (meniscal or cartilage) at primary or revision ACLR was not an exclusion criterion. If patients underwent knee arthroplasty or osteotomy at follow-up they were excluded.

All patients underwent surgery at the same clinic using a single-bundle autologous HT. The triple or quadruple semitendinosus tendon or semitendinosus and gracilis tendons were used. For revision surgery the BPTB graft was harvested as the central third of the patellar tendon with two bone blocks. The femoral tunnel was drilled using an anteromedial portal technique. In the majority of cases, the grafts were fixed using an EndoButton fixation device (Smith & Nephew, Andover, MA) or an interference screw on the femoral side and No. 2 Ethibond sutures (Ethicon, Somerville, NJ) tied over an AO bi-cortical screw with a washer as a post or using an interference screw on the tibial side. No lateral extraarticular surgery was done in any of the patients.

The preoperative KOOS, TAS and surgical data for the included patients were extracted from the database. To collect data at follow-up, a letter was sent including a QR-code to the questionnaires KOOS and EuroQol 5-dimensions (EQ-5D). A week after sending the letter, patients were contacted by phone and asked a questionnaire consisting of study-specific questions, which were adapted from the questionnaire proposed by Koca et al. [16] to suit revision ACLR patients. During the telephone call, patients were also reminded to answer the KOOS and EQ-5D questionnaires. The KOOS and EQ-5D obtained at follow-up were compared with the preoperative KOOS and EQ-5D recorded in our database.

The patients who responded to the telephone questionnaire were subsequently paired with patients with primary ACLR, who formed the control group using 1:1 matching. The revision ACLR group and the control group (primary ACLR) were matched based on sex, age (± 2 years), year of ACLR, (all at the time of the second ACLR for the revision group), and type of activity (TAS) at the time of the primary ACL injury. As an example, a male football player born in 1998, who had a revision ACLR in 2014 was paired with another male football player born in 1998 (within a range of ± 2 years), who underwent primary ACLR in the same year of 2014. If a male football player was unavailable, a male athlete engaged in a sport sharing the same TAS and featuring a comparable level of pivoting activity, such as floorball, basketball, or, handball was selected as a substitute. When the matched control was found, the primary and revision ACLR patients were administered an identical questionnaire, with the exception that the study-specific questions pertaining to the revision ACLR were excluded for the primary ACLR group. This involved the removal of questions regarding activity level following the revision ACLR and activities performed at the revision ACL injury. If the matched control did not respond within a period of two weeks, that individual was excluded from the study and a new matched control was identified.

### Data collection

#### Patient-reported outcomes and study-specific questionnaires

The KOOS consists of five subscales with possible scores ranging from 0 (worse) to 100 (best): Symptoms, Pain, Activities of Daily Living (ADL), Sport and Recreation (Sports/Recreation) and Knee-related Quality of Life (QOL) [14]. The attainment of a patient-acceptable symptom state, as defined by Roos et al. [17], was evaluated using predetermined threshold values: Pain (≥ 89), Symptoms (≥ 83), ADL (≥ 95), Sports/Recreation (≥ 72) and QOL (≥ 73).

The EQ-5D is a standardized measure used to assess health-related quality of life [18]. The scores are converted into index values, ranging from less than 0 (indicating the worst health state) to 1 (representing the best health state). Additionally, the EQ-VAS allows individuals to subjectively rate their own health status on a vertical VAS ranging from 0 (worst imaginable health state) to 100 (best imaginable health state).

The questionnaire designed specifically for this study was administered during the telephone call. It collected information about the participants’ occupation, including any occupational changes resulting from their ACL injury. Additionally, the questionnaire asked patients to report about any history of ACL injuries in the family, the activity level prior to the first ACL injury, and the activity levels following both the initial ACLR and the subsequent revision ACLR for the revision group. The patients reported their activity levels and were graded by the authors according to an updated version of the TAS with a range of 0 to 10, with 0 denoting the least demanding activities for the knee and 10 indicating activity on an elite level, which involves the highest level of exertion [15, 19]. The activity level was also graded according to the guidelines from the World Health Organisation (WHO) on physical activity [20] using the questions “How much time did you spend last week 1) on everyday exercise, e.g. cycling, walking, or gardening? and 2) doing physical exercise that makes you short of breath, e.g. running, gymnastics or ball sports?” with fixed response options. Patients were additionally requested to indicate their satisfaction level with regard to their current activity level with a scale ranging from 1 (not satisfied at all) to 10 (very satisfied). The question “Do you feel limited when exercising after your ACLR on a 10-point scale?”, where 1 corresponded to “very limited” and 10 corresponded to “not limited at all”, was used to rate limitation in sport [16]. Current global knee function was evaluated with the question “How would you rate your knee function on a 10-point scale?”, where 0 corresponded to “inability to cope with normal daily activities” and 10 corresponded to “normal, excellent function” from the International Knee Documentation Committee-Subjective Knee Form (IKDC-SKF) [21]. Knee satisfaction was assessed using a 10-point scale, where a rating of 1 indicated “not satisfied at all” and a rating of 10 corresponded to “very satisfied” [15, 16]. The period of time taken for patients to RTS after undergoing primary ACLR was documented, and they were also asked about any factors or circumstances that prevented them from RTS.

### Statistical analysis

The Statistical Package for the Social Sciences (SPSS) version 27.0 (SPSS Inc., Chicago, Illinois) was used for all statistical analyses. Descriptive statistics were computed, including mean and standard deviation for ratio data, median, range and interquartile range (IQR) for interval data, and frequency and proportions for categorical data. A drop-out analysis regarding sex and age in the revision ACLR group was performed using the Pearson’s chi-square test. A paired-sample *t*-test was used for comparisons of time to RTS after primary ACLR, KOOS subscale scores, EQ-5D and EQ-5D VAS between the groups. The Wilcoxon Signed Rank test was utilized to compare the activity level and patients’ self-assessments on a 10-point scale. The McNemar’s test was used for comparisons between the revision ACLR and primary ACLR group regarding the proportion of patients who changed occupation, educational level, had a family history of ACL injury, achieved a patient-acceptable symptom state, reached the WHO’s recommendation of physical activity, RTS and returned to the same level of sport. A paired-sample t-test was used for comparisons of KOOS subscale scores within groups (primary and revision ACLR). The level of significance was set at *p* < 0.05 for all analyses.

## Results

### Patients

**Fig. 1:**
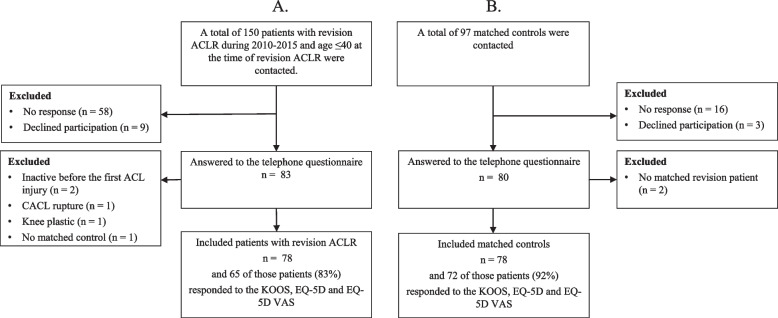
Flow chart of patient enrolment. **A** Revision ACLR Group and **B** Primary ACLR group. *ACLR*, anterior cruciate ligament reconstruction, *CACL*, contralateral anterior cruciate ligament

A total of 150 patients who underwent revision ACLR, were identified. Eighty-three patients (55%) answered the telephone questionnaire and 78 of these were included (Fig. 1). A drop-out analysis showed that significantly more women answered compared with men (58% vs 42%, *p* = 0.023). Of the 78 included patients, 65 (83%) responded to the KOOS, EQ-5D and EQ-5D VAS. The median time between the primary ACLR and the subsequent revision ACLR was 26 (IQR 16–40) months. The mean postoperative follow-up from the revision ACLR was 7.0 ± 1.5 years (range 4.2 − 10.1).

In all, a letter was sent to 97 patients (because of some drop-outs) for the control group to match the 78 revision ACLR patients. Out of the total participants, 80 individuals responded and provided informed consent, however two of them were excluded as their corresponding matched revision patient was excluded afterwards due to a total knee replacement surgery and a contralateral ACL (CACL) rupture. The mean postoperative follow-up for patients with a primary ACLR was 7.7 ± 1.6 years (range 4.9 − 12.3).

### Baseline characteristics

Patients’ characteristics as well as injury and surgery characteristics are shown in Tables 1 and 2. A family history of ACL injury was significantly more common in the revision ACLR group (40% vs 23%; *p* = 0.024) (Table 1). Football and downhill skiing were identified as the two most frequently reported activities at the time of ACL injury for both the revision ACLR group (both first and second ACL injury) and the primary ACLR group. Forty-eight of the patients (68%) were matched exactly to the same sport performed.

**Table 1:** Patient characteristics in the revision and primary ACLR groups

	Revision ACLR group (*n* = 78)	Primary ACLR group (*n* = 78)	Mean difference (95% CI)	*P*
Sex, male/female	33/45 (42/58)	33/45 (42/58)		
Age at follow-up, mean ± SD, y	29.9 ± 6.0	30.2 ± 5.8	-0.3 (-0.7 − 0.0)	0.079
Educational level				0.564
Low (0–9 years)	1 (1)	0 (0)		
Medium (10–14 years)	33 (42)	31 (40)		
High (≥ 15 years)	44 (56)	47 (60)		
Occupation				
Changed occupation due to the knee injury	9 (12)	3 (4)		0.083
Worker, mainly sedentary	41 (53)	36 (46)		0.637
Worker, mainly physical	23 (29)	30 (39)		
Student	14 (18)	12 (15)		
Family history of ACL injury	31 (40)	18 (23)		**0.024**

Data are reported as n (%) unless otherwise stated

*ACL* anterior cruciate ligament, *ACLR* anterior cruciate ligament reconstruction, *SD* standard deviation

**Table 2:** Characteristics of injury and surgery in patients with revision and primary ACLR

	Revision ACLR group (*n* = 78)		Primary ACLR group (*n* = 78)
	Primary ACLR	Revision ACLR	Primary ACLR
Time between ACLR, median (IQR), months	26 (16–40)		
Index knee ACLR			
Right	40 (51)		38 (49)
Left	38 (49)		40 (51)
Patients with concomitant injuries	30 (39)^a^	22 (33)^b^	27 (35)
Concomitant injuries	36 (46)	26 (33)	31 (40)
Medial meniscal tear	13 (17)	7 (9)	8 (10)
Lateral meniscal tear	13 (17)	9 (12)	14 (18)
Medial and lateral meniscal tears	4 (5)	6 (8)	3 (4)
Chondral lesion	6 (8)	4 (5)	6 (8)
Age at ACLR, mean ± SD, y	20.3 ± 5.7	22.9 ± 6.0	22.5 ± 5.9
Males, mean ± SD, y	22.9 ± 6.3	25.3 ± 6.7	25.0 ± 6.6
Females, mean ± SD, y	18.4 ± 4.3	21.1 ± 4.7	20.6 ± 4.5
Time between ACLR and follow-up, mean ± SD, y	9.5 ± 1.9	7.0 ± 1.5	7.7 ± 1.6
Graft type, HT/BPTB autograft	71/3 (96/4)^c^	4/62 (6/94)^d^	71/7 (91/9)
Activity performed at injury			
Football	44 (56)	31 (40)	49 (63)
Downhill skiing	8 (10)	9 (12)	10 (13)
Handball	4 (5)	4 (5)	4 (5)
Basketball	4 (5)	4 (5)	2 (3)
American football	4 (5)	2 (3)	2 (3)
Floorball	3 (4)	2 (3)	3 (4)
Other	11 (14)	26 (33)	8 (10)

Data are reported as n (%) unless otherwise stated

*ACL* anterior cruciate ligament, *ACLR* anterior cruciate ligament reconstruction, *BPTB* bone-patellar tendon-bone, *HT* hamstring tendon, *IQR* interquartile range, *SD* standard deviation

^a^2 missing answers

^b^9 missing answers

^c^4 missing answers

^d^12 missing answers

### Subjective knee function and activity level

Patients with a revision ACLR rated their knee function lower, were less satisfied with their ACLR knee, were less physically active with everyday exercises, felt more limited when exercising and had lower scores on the EQ-VAS compared with patients with a primary ACLR (*p* < 0.05, Table 3). No differences between the groups on the EQ-5D, activity level or physical exercises and satisfaction with activity level after primary ACLR and at follow-up were found (Table 3). Following the initial ACLR, 73% of the patients in the revision ACLR group successfully resumed participation in the same sport, while 53% were able to return to their preinjury activity level. In comparison, the primary ACLR group demonstrated similar rates, with 62% of the patients returning to same sport (*p* = 0.182) and 51% achieving the same preinjury activity level (*p* = 0.739). Among those who returned to their previous activity level, patients in the revision ACLR group returned significantly earlier than patients in the primary ACLR group (9.6 vs 12.7 months, *p* < 0.001). Following revision ACLR, 45% of the patients were able to resume participation in the same sport, while 26% achieved their preinjury activity level. The predominant reasons reported by patients in both groups for not achieving their preinjury activity level were fear of experiencing another injury and impaired knee function (Table 4).

**Table 3:** Answers from the telephone questionnaire and EQ-5D from the revision ACLR (*n* = 78) and primary ACLR groups (*n* = 78) at follow-up

	Revision ACLR group	Primary ACLR group	Mean difference (95% CI)	*p*-value
Knee function (0–10)	7 (2–10; 6–8)	8 (3–10; 7–9)		** < 0.001**
Satisfaction with knee function (1–10)				
ACLR knee	7 (1–10; 6–8)	8 (4–10; 7–9)		** < 0.001**
Uninjured knee	10 (1–10; 9–10)	10 (5–10; 10–10)		0.050
I feel limited when exercising (1–10)	7 (1–10; 4–8)	8 (4–10; 6–9)		** < 0.001**
Satisfaction with activity level (1–10)	8 (1–10; 5–9)	8 (1–10; 6–10)		0.281
Tegner Activity Scale				
Before first injury	9 (2–10; 8–9)	9 (2–10; 7–9)		0.177
After first ACLR	8 (2–10; 6–9)	7 (1–10; 3–9)		0.067
After second ACLR	5 (1–10; 2–8)			
At follow-up	2 (1–10; 2–5)	4 (1–10; 2–7)		0.229
Time last week (at follow-up) for:				
Everyday activities, minutes^a^				**0.003**
0	2 (3)	1 (1)		
< 30	1 (1)	0 (0)		
30–60	4 (5)	1 (1)		
60–90	5 (6)	2 (3)		
90–150	12 (15)	8 (10)		
150–300	17 (22)	10 (13)		
> 300	37 (47)	55 (71)		
Physical activities, minutes^b^				0.103
0	12 (15)	14 (19)		
< 30	0 (0)	3 (4)		
30–60	2 (3)	4 (5)		
60–90	3 (4)	4 (5)		
90–120	4 (5)	5 (7)		
> 120	57 (73)	45 (60)		
Reaching the WHO’s recommendation for physical activity, n (%)^c^	74 (95)	74 (96)		0.705
EQ-5D (0–1)^d^	0.86 ± 0.17 (0.82–0.91)	0.89 ± 0.11 (0.87–0.92)	-0.02 (-0.07 − 0.03)	0.427
EQ-5D VAS (0–100)^d^	79.2 ± 20.1 (74.2–84.3)	86.0 ± 11.6 (83.3–88.8)	-7.5 (-13.3 − 1.7)	**0.012**

Data are presented as the mean ± SD (95% CI), median (range; interquartile range) or n (%)

*ACLR* anterior cruciate ligament reconstruction, *EQ-5D* EuroQol 5-Dimensions, *VAS* visual analogue scale

^a﻿^The question; “How much time did you spend last week on everyday exercise, e.g. cycling, walking, or gardening?”

^b﻿^The question; “How much time did you spend last week doing physical exercise that makes you short of breath, e.g. running, gymnastics or ball sports?”. The primary group, one missing answer

^c﻿^At least 150–300 min of moderate-intensity or at least 75–150 min of vigorous-intensity aerobic physical activity

^d﻿^The revision group, 16 missing answers. The primary group, seven missing answers

**Table 4:** Reasons for not returning to preinjury activity level

	Revision ACLR group (*n* = 57)	Primary ACLR group (*n* = 32)
Poor knee function	12 (21)	6 (19)
Not trusting the knee	6 (11)	3 (9)
Fear of suffering another injury	17 (30)	10 (31)
Change in team	3 (5)	4 (13)
Family situation	3 (5)	2 (6)
Work situation	0 (0)	1 (3)
Dissuaded by doctor/physiotherapist	8 (14)	1 (3)
Other	8 (14)	5 (16)

Number of patients and percentage of the group that did not return to their preinjury activity level

*ACLR* anterior cruciate ligament reconstruction

No significant differences between the groups regarding preoperative KOOS subscale scores were found. The patients with revision ACLR reported significantly better KOOS subscale scores preoperatively at their revision ACLR compared with their preoperatively primary ACLR (Table 5).

**Table 5:** Knee injury and osteoarthritis outcome score subscale scores for the revision ACLR group and primary ACLR group preoperative and at follow-up

	Knee injury and Osteoarthritis Outcome Score				
	Pain	Symptoms	ADL	Sport and Recreation	Knee-related QoL
**Preoperative**					
1. Revision ACLR group, primary ACLR (*n* = 70)	82.3 (13.8)	77.8 (15.3)	91.1 (9.9)	48.8 (29.2)	36.3 (23.5)
2. Revision ACLR group, second ACLR (*n* = 65)	86.6 (11.6)	82.1 (14.1)	94.2 (7.5)	60.2 (28.8)	46.2 (27.2)
Mean with-in difference (95% CI)					
1 compared to 2	-4.6 (-8.1 to -1.0)	-5.2 (-9.7 to -0.6)	-3.6 (-6.1 to -1.2)	-12.7 (-21.1 to -4.2)	-11.4 (-18.0 to -4.7)
*p*-value	**0.012**	**0.027**	**0.004**	**0.004**	**0.001**
3. Primary ACLR group, (*n* = 72)	81.8 (14.6)	77.8 (18.2)	90.5 (11.8)	55.6 (27.1)	41.0 (22.9)
Mean group difference (95% CI)					
1 compared to 3	0.8 (-3.2 to 4.9)	2.2 (-3.0 to 7.4)	0.9 (-2.2 to 4.1)	-6.3 (-14.3 to 1.6)	-5.3 (-13.3 to 2.5)
*p*-value	0.686	0.403	0.553	0.113	0.180
2 compared to 3	5.3 (1.0 to 9.5)	6.1 (0.2 to 12.0)	4.0 (0.7 to 7.4)	5.5 (-4.2 to 15.3)	4.6 (-4.7 to 13.9)
*p*-value	**0.017**	**0.044**	**0.020**	0.259	0.326
**Follow-up**					
4. Revision ACLR group (*n* = 65)	85.3 (12.6)	81.5 (14.1)	93.8 (7.7)	67.6 (27.0)	58.9 (20.8)
Mean with-in difference (95% CI)					
1 compared to 4	-2.3 (-6.6 to 2.0)	-2.5 (-7.8 to 2.9)	-1.9 (-4.8 to 1.0)	-17.7 (-27.2 to -8.2)	-20.5 (-28.8 to -12.1)
*p*-value	0.280	0.366	0.182	** < 0.001**	** < 0.001**
2 compared to 4	1.0 (-3.3 to 5.2)	1.6 (-3.4 to 6.6)	0.3 (-2.3 to 3.0)	-5.9 (-15.3 to 3.5)	-12.8 (-21.9 to -3.8)
*p*-value	0.643	0.526	0.799	0.212	**0.006**
5. Primary ACLR group (*n* = 72)	90.6 (11.2)	84.4 (15.2)	96.3 (7.4)	81.1 (18.4)	72.7 (19.7)
Mean with-in difference (95% CI)					
3 compared to 5	-8.5 (-12.9 to -4.1)	-7.9 (-13.1 to -2.6)	-5.5 (-8.6 to -2.4)	-25.6 (-32.8 to -18.4)	-32.2 (-39.9 to -24.5)
*p*-value	** < 0.001**	**0.004**	** < 0.001**	** < 0.001**	** < 0.001**
Mean group difference (95% CI)					
4 compared to 5	-5.9 (-10.0 to -1.8)	-3.2 (-8.1 to 1.8)	-3.3 (-5.5 to -1.2)	-13.7 (-21.0 to -6.3)	-14.8 (-20.1 to -8.6)
*p*-value	**0.006**	0.203	**0.003**	** < 0.001**	** < 0.001**
Change in KOOS score preoperative to follow-up					
Mean group difference (95% CI)					
(a compared to d) – (c compared to e)	-8.1 (-14.2 to -2.1)	-8.0 (-15.8 to -0.3)	-5.5 (-9.2 to -1.9)	-11.9 (-22.9 to -0.9)	-15.6 (-26.3 to -5.0)
*p*-value	**0.009**	**0.042**	**0.004**	**0.035**	**0.005**

*ACLR* anterior cruciate ligament reconstruction, *ADL* Activities of Daily Living, *QOL* Quality of Life

^a^Data are presented as the mean ± SD unless otherwise indicated

The primary ACLR group exhibited significantly greater improvements from pre-operatively to post-operatively in all subscales of KOOS compared with the revision ACLR group (Table 5). The improvement within the revision ACLR group from preoperatively for the primary ACLR to follow-up was significantly greater for the Sports/Recreation and KOOS QOL subscale scores (*p* < 0.001). The improvement within the revision ACLR group from preoperatively for the revision ACLR to follow-up was significantly greater only for the KOOS QOL subscale score (*p* = 0.006). The primary ACLR group demonstrated a significantly greater improvement across all the subscales of the KOOS from preoperative assessment to the follow-up (all *p* < 0.001, except Symptoms *p* = 0.004).

**Fig. 2:**
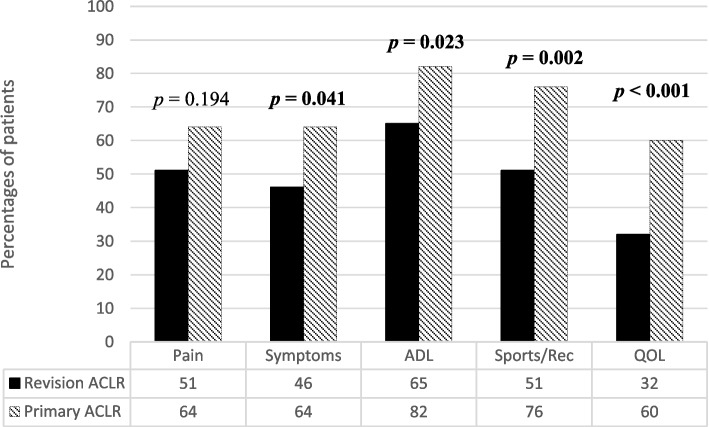
The proportion of patients attaining a patient-acceptable symptom state on the KOOS subscales during follow-up was examined in both the revision ACLR group (*n* = 65) and the primary ACLR group (*n* = 72). *ACLR* anterior cruciate ligament reconstruction, *ADL* Activities of Daily Living, *KOOS* Knee injury and Osteoarthritis Outcome Score, *QOL* Quality of Life, *Sports/Rec* Sports/Recreation

At the follow-up assessment, the patients in the primary ACLR group achieved a patient-acceptable symptom state across all KOOS subscales to a higher degree in comparison with patients in the revision ACLR group, except for the KOOS Pain subscale (Fig. 2).

## Discussion

The most important finding in the present study was that the revision ACLR group reported poorer knee function and quality of life, less satisfaction with knee function and greater limitation in sport but no differences in activity level and satisfaction with activity level compared with the primary ACLR group at a minimum 5-year follow-up.

Patients with revision ACLR reported poorer knee function in KOOS, as well as in the 10-point scale evaluating knee function and satisfaction with knee function, but not in the EQ-5D compared with patients with primary ACLR. Moreover, the improvement in all KOOS subscales from preoperative to postoperative was smaller compared with that of patients with primary ACLR. The largest and only clinical relevant (8–10 points) [22] differences were reported in the Sports/Recreation and QoL subscales (13–14 points). However, the proportion of patients attaining a patient-acceptable symptom state on all the KOOS subscales were lower for the revision group compared with patients with primary ACLR. The Pain, Sports/Recreation and QoL subscales have previously been reported to be the most sensitive to changes in the condition of the knee [14]. Poorer KOOS scores in patients with revision ACLR compared with patients with primary ACLR at a follow-up of 2 to 9 years were previously reported [23]. A meta-analysis with a minimum of 2 years of follow-up comparing patient-reported outcomes measured with the Lysholm score reported that patients with revision ACLR had inferior scores compared with patients with primary ACLR [10]. However, the same meta-analysis [10] reported that there were insufficient data of patient-reported scores other than the Lysholm score, such as the IKDC-SKF or the KOOS. In contrast, a previous matched-group analysis based on 63 patients with revision ACLR and primary ACLR was not able to find significant differences in the Lysholm score between the groups at 3 to 5 years of follow-up [13]. A clinical review from 2019 [11] concluded that patients with revision ACLR have poorer patient-reported outcomes, but most studies evaluating patient-reported outcomes comparing revision and primary ACLR have small patient samples, a short follow-up and are mostly retrospective. Patients with revision ACLR may have poorer patient-reported knee function and quality of life than patients with primary ACLR, because they have had to undergo two ACLRs that imply harvesting most often both HT and BPTB grafts and they also have more concomitant injuries to the menisci and cartilage [9, 24].

When analyzing within-group differences, the revision ACLR group reported better preoperative KOOS scores in all subscales at the time of revision ACLR compared with their primary ACLR, with the largest and only clinically relevant [22] difference in the Sport/Recreation and QoL subscales. This is in accordance with the findings of a recent study [7] in which the authors hypothesised that the ACL re-injury may have less impact on the perceived life situation compared with the first ACL injury. The improvement in KOOS scores from preoperatively (at both the primary and revision ACLR) to follow-up for the revision ACLR group was significantly greater only in the KOOS Sports/Recreation and QOL subscales. Preoperatively, at the revision ACLR, 33% of the patients had concomitant injuries. The additional injuries could have affected the outcome.

For the primary ACLR group all the KOOS subscales improved significantly, but was only clinically relevant for Sport/Recreation and QoL subscales [22]. However, the KOOS sub scores Sport/Recreation and knee-related QoL are considered having the greatest room for improvement and highest content validity compared with other KOOS subscales [25].

Health-related quality of life assessment with EQ-5D did not differ between the groups and was similar to the results reported in a systematic review with a meta-analysis of long-term reports of patients with ACLRs [26]. In comparison to the primary ACLR group, the revision ACLR group reported a lower rating on the EQ-5D VAS (79 vs 86). However, previously reported EQ-5D VAS measured one and two years after primary ACLR showed results similar to those in our revision ACLR group [27].

Activity level, except everyday exercises, RTS and satisfaction with activity level at follow-up, did not differ between patients with revision ACLR and primary ACLR. Patients with revision ACLR returned to sport earlier after primary ACLR and reported greater limitation in sports. Return to a high activity level [28] and early RTS [29] have been shown to be risk factors for sustaining a new ACL injury and could be the reason for the ACL graft rupture in our cohort. Both groups reduced their activity level according to the TAS. The TAS decreases over time as numerous female football players progressively cease their involvement in knee-intensive activities due to several factors, such as familial and occupational obligations [30]. However, most of the patients in both groups fulfilled the recommendations for physical activity according to the WHO guidelines for adults (at least 150–300 min of moderate-intensity or at least 75–150 min of vigorous-intensity aerobic physical activity) for substantial health benefits and sedentary behaviour on the amount of physical activity [20]. A previous study comparing patients with revision ACLR and primary ACLR regarding RTS at nine months after surgery reported that athletes with primary ACLR were more likely to have returned to sport [31]. Another study, comparing athletes with revision ACLR with matched athletes with primary ACLR, reported that athletes with primary ACLR were more likely to RTS overall (84% vs 65%), but there were no differences between the groups regarding the return to the same activity level (56% vs 49%) 3 to 5 years of follow-up [13]. In our study, the main reasons for not RTS were fear of re-injury in both groups. Fear of re-injury is a common reported cause of quitting sport after ACLR [4, 15, 16, 32]. A previous study reported that patients with revision ACLR quit sports because of a fear of re-injury to a higher degree compared with athletes with primary ACLR and this was the most common factor that caused changes in sports activity in athletes with revision ACLR [13].

In the present study, more patients in the revision ACLR group reported familial history for an ACL injury (40% vs 23%). A recent review showed that a family history of ACL injury increases the odds (more than double) of sustaining a primary or additional ACL injury [33] and this has also been reported as a risk factor for revision surgery [28]. This information could be important when it comes to identifying patients at risk of ACL re-rupture and eventual revision ACLR.

One strength is the homogeneity of the study groups, as all ACLRs were performed at the same clinic, employing a standardized surgical technique and postoperative rehabilitation protocol. The telephone interview at a minimum 5-year follow-up deepened the understanding of RTS regarding level pre- and post ACLR. Some limitations should be acknowledged. The study included a relatively small sample size with a cross-sectional design. No clinical evaluation was performed providing objective data concerning knee status as e.g. range of motion and knee laxity. The response rate to the telephone questionnaire was only 55%, with more women answering. However, cross-sectional survey studies of patients with ACLR distributed by mail generally have a low response rate (38–59%) [32, 34], with more women answering [34]. Another limitation of the study is the reliance on patients’ recall of their activity and activity level, which introduces the potential for recall bias. Moreover, activity and activity level in active patients who sustained an ACL injury are an important life event and recall bias should be a minor problem. Patient reported activity level could be overestimated, but this problem should be equal between the groups. The reference values for the KOOS patient-acceptable symptom state are from patients with primary ACLR and could differ in patients with subsequent ACLR. The study groups were matched by sex, age, year of ACLR and preinjury sport, but, in some cases, the patients were not matched to exactly the same sport. However, in these cases, the patients were matched by a comparable level of pivoting activity, as determined by the TAS. No matching for meniscus or cartilage injuries was performed. However, both groups had a similar rate of concomitant injuries at baseline.

## Conclusion

Patients with revision ACLR reported poorer knee function and quality of life, less satisfaction with knee function and a greater limitation in sports but no differences in activity level and satisfaction with activity level compared with patients with primary ACLR. This information can help clinicians in counseling patients about the long-term outcomes following revision ACLR in comparison with primary ACLR.

## Data Availability

All raw data and materials utilized during the study are available from the corresponding author by reasonable request.

## Abbreviations

ACL: Anterior Cruciate Ligament
ACLR: Anterior Cruciate Ligament Reconstruction
ADL: Activities of Daily Living
BPTB: Bone-patellar tendon-bone
CACL: Contralateral anterior cruciate ligament
HT: Hamstring tendon
IQR: Interquartile range
IKDC-SKF: International Knee Documentation Committee Subjective Knee Form
KOOS: Knee injury and Osteoarthritis Outcome Score
PROMs: Patient-reported outcome measurements
RTS: Return To Sport
QOL: Quality of Life
SD: Standard deviation
Sports/Rec: Sports/Recreation
WHO: World Health Organisation
